# Tricuspid annular and right atrial volume changes are associated in healthy adults—insights from the three-dimensional speckle-tracking echocardiographic MAGYAR-Healthy Study

**DOI:** 10.3389/fcvm.2023.1140599

**Published:** 2023-09-04

**Authors:** Attila Nemes, Árpád Kormányos, Gergely Rácz, Zoltán Ruzsa, Alexandru Achim, Nóra Ambrus, Csaba Lengyel

**Affiliations:** Department of Medicine, Albert Szent-Györgyi Medical School, University of Szeged, Szeged, Hungary

**Keywords:** healthy, tricuspid annulus, right atrial, volume, three-dimensional, speckle-tracking, echocardiography

## Abstract

**Introduction:**

The tricuspid valve and its annulus (TA) and the right atrium (RA) play a significant role in regulating blood flow in the right heart. However, their effect on each other is not fully understood even in normal circumstances. Three-dimensional (3D) speckle-tracking echocardiography (3DSTE) is able to simultaneously assess TA and RA at the same time in a non-invasive way. The present study aimed to examine associations between tricuspid annular (TA) dimensions and right atrial (RA) volumes in healthy adults by 3DSTE.

**Methods:**

The present study comprised 144 healthy subjects (mean age: 34.4 ± 12.6 years, 72 males), who participated in this study on a voluntary basis for screening between 2011 and 2015. In all subjects, electrocardiography, two-dimensional Doppler echocardiography and 3DSTE have been performed.

**Results:**

With increasing end-systolic maximum RA volume, all end-systolic and end-diastolic TA dimensions showed simultaneous increase, but in various degrees resulting in (non-significant) reduction of TA functional properties. Similarly, with increasing diastolic pre-atrial contraction and minimum RA volumes, TA dimensions increased simultaneously (except end-diastolic TA diameter), but in various degrees resulting in reduced TA fractional shortening and fractional area change. With increasing RA dimensions, end-systolic and end-diastolic TA dimensions showed simultaneous increase, but in different, sometimes not significant degrees. While RA stroke volumes showed increasing pattern with TA dilation, RA emptying fractions have not changed substantially.

**Conclusions:**

3DSTE is suitable for non-invasive assessment of TA dimensions and RA volumes at the same time using the same 3D echocardiographic dataset. Significant associations between TA size and RA volumes exist in healthy circumstances. Strong associations in case of dilation of TA in the presence of higher RA volumes could partly explain functional tricuspid regurgitation later developing in subjects in sinus rhythm.

## Introduction

In recent years, there has been an increasing interest in the evaluation of the right heart due to newer therapeutic options and advanced imaging techniques (1–4). New findings help to better understand the right heart, its components, their characteristics and interactions, even with the aorta, left heart and venous system, and their dependence on each other (1–4). The tricuspid valve (TV) and its annulus (TA) and the right atrium (RA) play a significant role in regulating blood flow in the right heart. Their effect on each other was examined in subjects with functional tricuspid regurgitation (FTR) (5–7) and even in healthy normal circumstances (8, 9). Three-dimensional speckle-tracking echocardiography (3DSTE) is able to simultaneously assess TA and RA in detail at the same time in a non-invasive way (10–12). The present study aimed to examine associations between tricuspid annular (TA) dimensions and right atrial (RA) volumes respecting the cardiac cycle in healthy adults by 3DSTE.

## Patients and methods

### Study population

The present study comprised 144 healthy subjects (mean age: 34.4 ± 12.6 years, 72 males), who participated in this study on a voluntary basis for screening between 2011 and 2015. In all subjects, electrocardiography (ECG), two-dimensional Doppler echocardiography (2DE) and 3DSTE have been performed by the same observer (ÁK). A participant was considered to be healthy if they had no acute or chronic illness in their medical history, ECG showed no abnormality, and findings of complete 2DE were in normal ranges. None of the subjects were obese, smoker or had a history of regular drug use. The present study is part of the **M**otion **A**nalysis of the heart and **G**reat vessels b**Y** three-dimension**A**l speckle-t**R**acking echocardiography in **Healthy** subjects (**MAGYAR-Healthy**) **Study**. This study aimed to evaluate the physiological associations among 3DSTE-derived and other parameters in healthy adults (“Magyar” means “Hungarian” in Hungarian language). The study was conducted in accordance with the Declaration of Helsinki (as revised in 2013). The study was approved by the Institutional and Regional Human Biomedical Research Committee of University of Szeged, Hungary (No.: 71/2011 and updated versions) and informed consent was given by all subjects.

### Two-dimensional Doppler echocardiography

In all cases, the same Toshiba Artida^TM^ echocardiography equipment (Toshiba Medical Systems, Tokyo, Japan) was used attached to a 1–5 MHz PST-30BT phased-array transducer. During chamber quantifications the rules prescribed in recent guidelines were followed (1). For visual quantification of valvular regurgitations and to exclude significant valvular stenosis, Doppler echocardiography was used. Early and late mitral inflow E and A were also determined to assess LV diastolic function (1, 13).

### Three-dimensional speckle-tracking echocardiography

The same Toshiba Artida^TM^ echocardiographic equipment (Toshiba Medical Systems, Tokyo, Japan) was used for 3DSTE as well, but transducer was changed to a PST-25SX matrix-array transducer with 3DSTE capability (10–12, 14, 15). The protocol of the 3DSTE examination followed our routines: firstly, 3D echocardiographic datasets were acquired from the apical window following optimalisation of image quality on the right atrium (RA). If RR intervals were constant on ECG (sinus rhythm) and subjects were on breath-hold, pyramid-shaped 3D echocardiographic datasets were digitally stored on hard drive for future analysis.

### RA-quantification by 3DSTE

**Figure 1:**
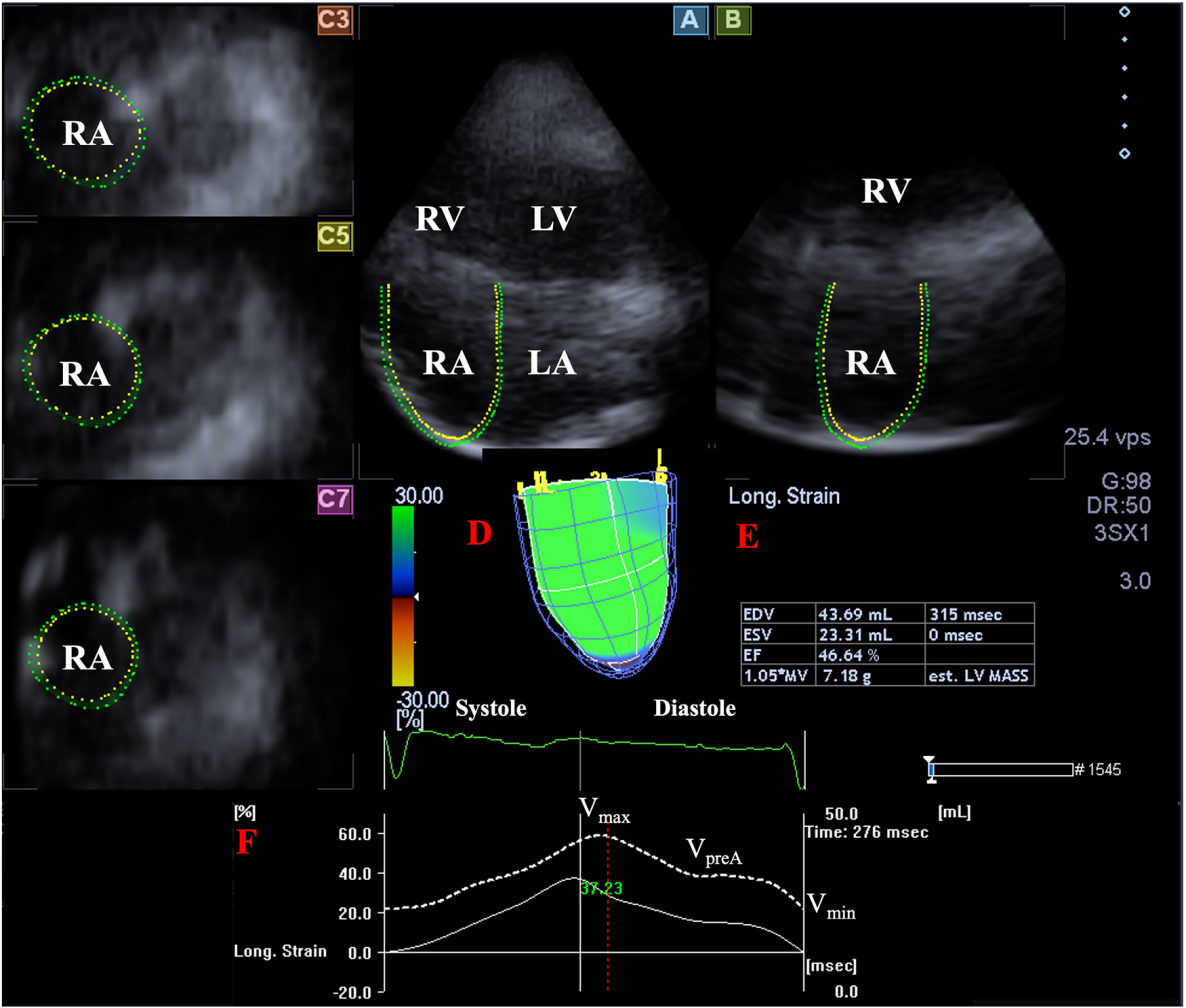
Three-dimensional (3D) speckle-tracking echocardiographic analysis of the right atrium in a healthy subject: apical longitudinal four-chamber (**A**) and two-chamber views (**B)** and 3 short-axis views at basal (**C**3), midatrial (**C**5) and superior (**C**7) RA levels. 3D virtual RA model (**D**), RA volumetric data (**E**) and time—global RA volume change curve (dashed white curve) and time—global RA longitudinal strain curve (white curve) respecting the cardiac cycle are demonstrated (**F**). LA, left atrium; LV, left ventricle; RA, right atrium; RV, right ventricle; EDV, end-diastolic volume; ESV, end-systolic volume; EF, ejection fraction; est., estimated; MV, myocardial volume; V_max_, maximum right atrial volume; V_preA_, volume at the onset of atrial systole; V_min_, minimum right atrial volume.

Later, offline analysis was performed with the vendor-provided 3D Wall Motion Tracking software version 2.7 (Ultra Extend, Toshiba Medical Systems, Tokyo, Japan). Data were displayed in selected apical two- (AP2CH) and four-chamber (AP4CH) views and 3 short-axis views at basal, midatrial and superior levels. To create a 3D cast of the RA, definition of reference points on RA endocardium were required in AP2CH and AP4CH views on the edges of the TA ring and the RA apex at end-diastole, then automatic sequential analysis (reconstruction) was performed for the complete endocardial RA surface. Taking into account the cardiac cycle, the following RA volumes were obtained (Figure 1) (14):

– Maximum RA volume, measured at end-systole, just before tricuspid valve opening (Vmax).
– RA volume before atrial contraction, measured at early-diastole at the time of the P wave on the ECG (VpreA).
– Minimum RA volume measured at end-diastole, just before tricuspid valve closure (Vmin).

Using RA volumes, several stroke volumes (SV) and emptying fractions (EF) could be determined featuring different phases of RA function:

*Reservoir function:*

– Total Atrial Stroke Volume (TASV): Vmax−Vmin.
– Total Atrial Emptying Fraction (TAEF): TASV/Vmax × 100.

*Conduit function:*

– Passive Atrial Stroke Volume (PASV): Vmax−VpreA.
– Passive Atrial Emptying Fraction (PAEF): PASV/Vmax × 100.

*Active contraction:*

– Active Atrial Stroke Volume (AASV): VpreA−Vmin.
– Active Atrial Emptying Fraction (AAEF): AASV/VpreA × 100.

### TA-quantification by 3DSTE

**Figure 2:**
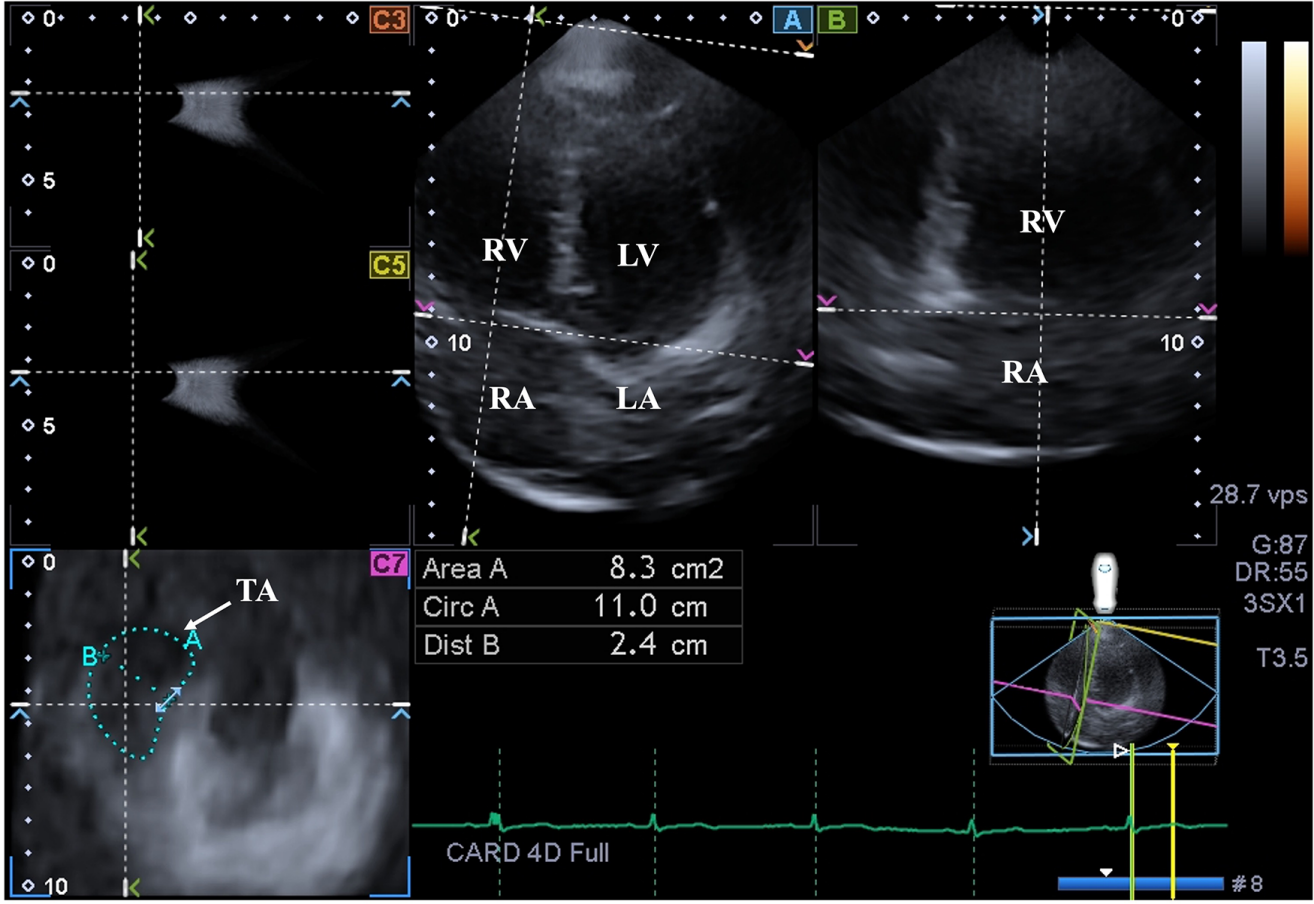
Three-dimensional (3D) speckle-tracking echocardiographic assessment of the tricuspid annulus in a healthy subject: apical longitudinal four-chamber (**A**) and two-chamber views and cross-sectional view (**C**7) of the tricuspid annulus optimalised on (**A**) and (**B**) images. White arrow indicate plane of the tricuspid annulus. LA, left atrium; LV, left ventricle; RA, right atrium; RV, right ventricle; Area, tricuspid annular area; Circ, tricuspid annular perimeter; Dist, tricuspid annular diameter.

During assessments, AP2CH and AP4CH views helped to find optimal lateral and septal TA endpoints on C7 short-axis view (Figure 2) (15):

*Morphological parameters* were measured at end-diastole (just before tricuspid valve closure) and at end-systole (just before tricuspid valve opening):

– TA diameter (TAD), measured by drawing a perpendicular line from the peak of TA curvature to the middle of the straight TA border,
– TA area (TAA), measured by planimetry,
– TA perimeter (TAP), measured by planimetry,

*Functional parameters* were calculated from morphologic end-diastolic and end-systolic parameters:

– TA fractional shortening (TAFS), defined as [(end-diastolic TAD—end-systolic TAD)/end-diastolic TAD] × 100,
– TA fractional area change (TAFAC), defined as [(end-diastolic TAA—end-systolic TAA)/end-diastolic TAA] × 100.

### Statistical analysis

Data were presented in mean ± standard deviation (SD) format or frequency/percentage format, as appropriate. *p *< 0.05 was considered to be statistically significant. Student *t* test with Welch correction and one-way analysis of variance (ANOVA) test with Bonferroni correction were used, where appropriated. Fischer's exact test was used for all categorical variables. Pearson's correlation coefficient was calculated for correlations. The Bland–Altman method was used to determine intraobserver and interobserver agreements. For intraobserver and interobserver correlations, intraclass correlation coefficients (ICCs) were calculated. Multivariable regression analysis was used for assessment of independent predictors of V_max_ and TAA-D. GPower 3.1.9 Software (Heinrich-Heine Universität, Düsseldorf, Germany) was applied for power calculation: in the presence of effect size: 0.9, alpha: 0.05, power: 0.8 the minimum group size is *n* = 120. Statistical calculations were performed using SPSS software (SPSS Inc, Chicago, IL, USA).

## Results

### Clinical and two-dimensional Doppler echocardiographic data

Clinical and routine echocardiographic parameters of healthy adults are presented in Table 1. None of the subjects involved had larger than or equal to grade 1 valvular regurgitation or showed significant valvular stenosis on any valves.

**Table 1:** Clinical and two-dimensional echocardiographic data.

Data	Measures
Clinical data	
*n*	144
Mean age (years)	34.4 ± 12.6
Males (%)	72 (50%)
Systolic blood pressure (mmHg)	121.4 ± 4.1
Diastolic blood pressure (mmHg)	78.9 ± 3.4
Heart rate (1/s)	71.3 ± 2.1
Height (cm)	170.8 ± 10.3
Weight (kg)	72.9 ± 15.5
Body surface area (kg/m^2^)	1.87 ± 0.36
Two-dimensional echocardiographic data	
LA diameter (mm)	37.8 ± 3.1
LV end-diastolic diameter (mm)	48.4 ± 3.1
LV end-systolic diameter (mm)	32.5 ± 2.6
LV end-diastolic volume (ml)	106.3 ± 23.3
LV end-systolic volume (ml)	38.2 ± 8.9
Interventricular septum (mm)	9.3 ± 1.1
LV posterior wall (mm)	9.4 ± 1.2
LV ejection fraction (%)	65.1 ± 3.5
Early diastolic mitral inflow velocity—E (cm/s)	79.4 ± 16.5
Late diastolic mitral inflow velocity—A (cm/s)	60.0 ± 15.0

LA, left atrial; LV, left ventricular.

### Classification of subjects

Mean ± SD of 3DSTE-derived RA and TA parameters of healthy subjects are presented in Table 2. Healthy subjects were classified into 3 groups according to the normal maximum (V_max_), pre-atrial contraction (V_preA_) and minimum (V_min_) RA volumes and end-systolic and end-diastolic TA diameter (TAD-S and TAD-D, respectively), area (TAA-S and TAA-D, respectively) and perimeter (TAP-S and TAP-D, respectively): estimated mean ± SD served as the lower (33.8 ml, 24.8 ml, 18.1 ml, 1.5 cm, 2.1 cm, 4.0 cm^2^, 5.7 cm^2^, 7.9 cm, 9.3 cm, respectively) and upper (64.0 ml, 46.8 ml, 37.5 ml, 2.1 cm, 2.7 cm, 6.8 cm^2^, 8.9 cm^2^, 10.1 cm, 11.7 cm, respectively) values.

**Table 2:** Three-dimensional speckle-tracking echocardiography-derived right atrial volumetric and tricuspid annular parameters.

Parameters	Measures
Maximum right atrial volume (V_max_, ml)	48.9 ± 15.1
Pre-atrial contraction left atrial volume (V_preA_, ml)	35.8 ± 11.0
Minimum left atrial volume (V_min_, ml)	27.8 ± 9.7
Total atrial stroke volume (TASV, ml)	21.1 ± 10.0
Total atrial emptying fraction (TAEF, %)	42.5 ± 12.6
Passive atrial stroke volume (PASV, ml)	13.1 ± 8.3
Passive atrial emptying fraction (PAEF, %)	25.8 ± 11.7
Active atrial stroke volume (AASV, ml)	8.0 ± 4.8
Active atrial emptying fraction (AAEF, %)	22.5 ± 11.6
End-systolic tricuspid annular diameter (TAD-S, mm)	1.8 ± 0.3
End-systolic tricuspid annular area (TAA-S, mm^2^)	5.4 ± 1.4
End-systolic tricuspid annular perimeter (TAP-S, mm)	9.0 ± 1.1
End-diastolic tricuspid annular diameter (TAD-D, mm)	2.4 ± 0.3
End-diastolic tricuspid annular area (TAA-D, mm^2^)	7.3 ± 1.6
End-diastolic tricuspid annular perimeter (TAP-D, mm)	10.5 ± 1.2
Tricuspid annular fractional shortening (TAFS, %)	21.4 ± 8.8
Tricuspid annular fractional area change (TAFAC, %)	26.4 ± 10.6

### Increase of RA volumes and TA

With increasing end-systolic V_max_, all end-systolic and end-diastolic TA dimensions showed simultaneous increase, but in various degrees resulting in (non-significant) reduction of TA functional properties. Similarly, with increasing diastolic V_preA_ and V_min_, TA dimensions increased simultaneously (except TAD-D), but in various degrees resulting in reduced TAFS and TAFAC (Table 3).

**Table 3:** Right atrial volumes and tricuspid annular parameters in different right atrial volume groups.

	V_max _≤ 33.8 ml (*n* = 26)	33.8 ml < V_max _< 64.0 ml (*n* = 92)	64.0 ml ≤ V_max_ (*n* = 26)	V_preA _≤ 24.8 ml (*n* = 23)	24.8 ml < V_preA _< 46.8 ml (*n* = 100)	46.8 ml ≤ V_preA_ (*n* = 21)	V_min _≤ 18.1 ml (*n* = 22)	18.1 ml < V_min _< 37.5 ml (*n* = 93)	37.5 ml ≤ V_min_ (*n* = 29)
V_max_ (ml)	29.6 ± 3.4	47.5 ± 7.5*	73.7 ± 9.1*^,^**	31.7 ± 7.9	48.6 ± 10.7^†^	70.9 ± 13.3^†^^,^^††^	34.4 ± 9.7	47.1 ± 11.3^‡^	65.6 ± 14.6^‡^^,^^‡‡^
V_preA_ (ml)	23.1 ± 4.1	35.1 ± 6.5*	50.5 ± 10.8*^,^**	21.4 ± 3.3	35.3 ± 5.5^†^	55.4 ± 8.4^†^^,^^††^	23.4 ± 6.2	33.9 ± 5.9^‡^	51.2 ± 9.7^‡^^,^^‡‡^
V_min_ (ml)	18.1 ± 3.7	27.0 ± 7.4*	39.8 ± 8.1*^,^**	16.0 ± 2.9	27.1 ± 6.2^†^	44.8 ± 4.5^†^^,^^††^	15.3 ± 2.3	25.9 ± 4.7^‡^	43.3 ± 4.5^‡^^,^^‡‡^
TASV (ml)	11.5 ± 3.1	20.4 ± 7.7*	33.9 ± 8.9*^,^**	15.7 ± 8.0	21.4 ± 9.3^†^	26.1 ± 12.3^†^	19.1 ± 9.3	21.2 ± 9.2	22.3 ± 12.7
TAEF (%)	38.9 ± 9.4	42.8 ± 13.6	45.9 ± 10.1*	47.4 ± 12.4	42.9 ± 12.5	35.1 ± 10.7^†^^,^^††^	52.9 ± 12.1	43.4 ± 10.3^‡^	31.7 ± 12.0^‡^^,^^‡‡^
PASV (ml)	6.6 ± 3.4	12.3 ± 6.3*	23.2 ± 9.6*^,^**	10.4 ± 7.2	13.3 ± 7.9	15.5 ± 11.0	11.0 ± 7.7	13.2 ± 7.8	14.4 ± 10.3
PAEF (%)	22.1 ± 11.0	25.5 ± 11.2	31.4 ± 12.3*^,^**	30.4 ± 13.9	25.8 ± 10.9	20.4 ± 11.8^†^^,^^††^	30.0 ± 15.3	26.4 ± 10.4	20.5 ± 11.4^‡^^,^^‡‡^
AASV (ml)	4.9 ± 2.2	8.1 ± 4.6*	10.8 ± 5.6*^,^**	5.3 ± 2.5	8.1 ± 4.5^†^	10.6 ± 6.2^†^^,^^††^	8.1 ± 5.0	8.0 ± 3.9	8.0 ± 6.8
AAEF (%)	21.2 ± 8.8	23.4 ± 13.0	20.9 ± 8.4	24.6 ± 10.2	23.0 ± 12.3	18.2 ± 8.5^†^	32.2 ± 12.9	23.0 ± 9.4^‡^	13.9 ± 10.9^‡^^,^^‡‡^
TAD-S (mm)	1.7 ± 0.3	1.8 ± 0.2*	2.0 ± 0.4 *^,^**	1.7 ± 0.3	1.8 ± 0.2^†^	2.0 ± 0.4^†^^,^^††^	1.7 ± 0.2	1.8 ± 0.2^‡^	1.9 ± 0.4^‡^
TAA-S (mm)	4.2 ± 1.2	5.4 ± 1.1*	6.6 ± 1.8*^,^**	4.4 ± 1.3	5.4 ± 1.2^†^	6.6 ± 1.9^†^^,^^††^	4.3 ± 1.3	5.4 ± 1.2^‡^	6.2 ± 1.8^‡^^,^^‡‡^
TAP-S (mm)	8.3 ± 1.2	9.0 ± 0.9*	9.9 ± 1.1*^,^**	8.5 ± 1.2	9.0 ± 1.0^†^	9.9 ± 1.2^†^^,^^††^	8.3 ± 1.2	9.0 ± 0.9^‡^	9.6 ± 1.2^‡^^,^^‡‡^
TAD-D (mm)	2.2 ± 0.3	2.3 ± 0.3*	2.5 ± 0.4*^,^**	2.3 ± 0.3	2.3 ± 0.3	2.5 ± 0.4	2.2 ± 0.4	2.4 ± 0.3	2.4 ± 0.4
TAA-D (mm)	6.0 ± 1.4	7.3 ± 1.3*	8.6 ± 1.7*^,^**	6.6 ± 1.7	7.3 ± 1.4^†^	8.6 ± 1.9^†^^,^^††^	6.3 ± 1.6	7.3 ± 1.4^‡^	8.2 ± 1.8^‡^^,^^‡‡^
TAP-D (mm)	9.5 ± 1.2	10.5 ± 1.0*	11.2 ± 1.0*^,^**	10.0 ± 1.5	10.4 ± 1.0	11.2 ± 1.1^†^^,^^††^	9.9 ± 1.3	10.4 ± 1.1^‡^	10.9 ± 1.1^‡^^,^^‡‡^
TAFS (%)	24.3 ± 10.1	21.1 ± 8.7	19.8 ± 7.0	26.1 ± 9.6	20.6 ± 8.5^†^	19.4 ± 8.0^†^	24.3 ± 10.8	21.4 ± 8.6	19.1 ± 7.4^‡^
TAFAC (%)	29.4 ± 12.3	26.5 ± 9.9	23.2 ± 10.5	32.5 ± 11.9	25.6 ± 10.1^†^	22.3 ± 9.0^†^	31.2 ± 11.8	25.9 ± 10.2^‡^	24.1 ± 10.0^‡^

V_max_, maximum end-systolic right atrial volume; V_preA_, early diastolic pre-atrial contraction right atrial volume; V_min_, minimum end-diastolic right atrial volume; TASV, total atrial stroke volume; TAEF, total (right) atrial emptying fraction; PASV, passive (right) atrial stroke volume; PAEF, passive (right) atrial emptying fraction; AASV, active (right) atrial stroke volume; AAEF, active (right) atrial emptying fraction; TAD, tricuspid annular diameter; TAA, tricuspid annular area; TAP, tricuspid annular perimeter; TAFS, tricuspid annular fractional shortening; TAFAC, tricuspid annular fractional area change; S, end-systolic; D, end-diastolic.

* *p* < 0.05 vs. V_max _≤ 33.8 ml.

** *p* < 0.05 vs. 33.8 ml < V_max _< 64.0 ml.

^†^ *p* < 0.05 vs. V_preA _≤ 24.8 ml.

^††^ *p* < 0.05 vs. 24.8 ml < V_preA _< 46.8 ml.

^‡^ *p* < 0.05 vs. V_min _≤ 18.1 ml.

^‡‡^ *p* < 0.05 vs. 18.1 ml < V_min _< 37.5 ml.

### Increase of RA volumes and RA volume-based functional properties

With increasing end-systolic V_max_, all SVs and EFs showed simultaneous increase, except AAEF, which remained unchanged. With increasing diastolic V_preA,_ while SVs showed increasing pattern, EFs had decreasing pattern. With increasing diastolic V_min,_ while SVs remained unchanged, EFs showed decreasing pattern (Table 3).

### Dilation of TA and RA volumes

With increasing TA dimensions, end-systolic and end-diastolic RA dimensions showed simultaneous increase, but in different degrees. While SVs showed increasing pattern with TA dilation, EFs have not changed substantially (Tables 4, 5).

**Table 4:** Right atrial volumes and tricuspid annular parameters in different end-systolic tricuspid annular groups.

	TAD-S_ _≤ 1.5 cm (*n* = 16)	1.5 cm < TAD-S < 2.1 cm (*n* = 102)	2.1 cm ≤ TAD-S (*n* = 26)	TAA-S_ _≤ 4.0 cm^2^ (*n* = 24)	4.0 cm < TAA-S < 6.8 cm^2^ (*n* = 103)	6.8 cm^2^ ≤ TAA-S (*n* = 17)	TAP-S_ _≤ 7.9 cm (*n* = 24)	7.9 cm < TAP-S < 10.1 cm (*n* = 102)	10.1 cm ≤ TAP-S (*n* = 18)
V_max_ (ml)	35.6 ± 10.4	49.5 ± 14.4*	54.9 ± 15.6*	35.3 ± 9.8	50.0 ± 13.3^†^	61.3 ± 18.4^†^^,^^††^	36.7 ± 10.5	50.0 ± 13.7^‡^	58.6 ± 18.6^‡^^,^^‡‡^
V_preA_ (ml)	27.0 ± 9.6	35.8 ± 10.1*	41.2 ± 11.9*^,^**	25.1 ± 6.9	36.5 ± 8.7^†^	46.2 ± 15.4^†^^,^^††^	27.6 ± 8.1	36.3 ± 9.4^‡^	43.4 ± 15.5^‡^^,^^‡‡^
V_min_ (ml)	21.3 ± 9.6	27.5 ± 9.0*	32.8 ± 9.8*^,^**	19.1 ± 6.6	28.5 ± 8.4^†^	36.0 ± 11.7^†^^,^^††^	20.7 ± 7.5	28.4 ± 8.9^‡^	34.0 ± 11.4^‡^^,^^‡‡^
TASV (ml)	14.3 ± 6.4	22.0 ± 9.9*	22.1 ± 10.6*	16.2 ± 9.1	21.5 ± 9.5^†^	25.3 ± 11.7^†^	16.0 ± 8.9	21.7 ± 9.7^‡^	24.6 ± 10.9^‡^
TAEF (%)	40.9 ± 15.2	48.7 ± 11.9	39.0 ± 13.1	44.7 ± 15.1	42.3 ± 11.9	40.4 ± 13.1	42.7 ± 14.7	42.7 ± 12.4	41.2 ± 11.4
PASV (ml)	8.6 ± 6.1	13.7 ± 8.2*	13.7 ± 9.1*	10.2 ± 8.2	13.5 ± 7.9	15.1 ± 10.4	9.0 ± 7.4	13.7 ± 8.1^‡^	15.1 ± 9.5^‡^
PAEF (%)	23.9 ± 15.5	26.6 ± 10.9	23.8 ± 12.3	27.1 ± 15.9	25.7 ± 10.2	24.2 ± 14.0	23.5 ± 15.2	26.4 ± 10.6	25.6 ± 12.9
AASV (ml)	5.7 ± 2.3	8.3 ± 4.7*	8.3 ± 5.8	6.0 ± 3.4	8.1 ± 4.6^†^	10.2 ± 6.2^†^	6.9 ± 3.7	8.0 ± 4.6	9.4 ± 6.4
AAEF (%)	22.7 ± 10.6	23.3 ± 11.6	19.8 ± 11.9	24.3 ± 11.2	22.3 ± 12.0	21.5 ± 9.4	25.5 ± 10.3	22.2 ± 12.1	20.8 ± 10.1
TAD-S (mm)	1.4 ± 0.1	1.8 ± 0.1*	2.3 ± 0.2*^,^**	1.6 ± 0.2	1.8 ± 0.2^†^	2.2 ± 0.3^†^^,^^††^	1.7 ± 0.2	1.8 ± 0.2^‡^	2.2 ± 0.4^‡^^,^^‡‡^
TAA-S (mm)	4.0 ± 0.9	5.2 ± 1.2*	6.8 ± 1.6*^,^**	3.5 ± 0.4	5.4 ± 0.8^†^	8.1 ± 1.3^†^^,^^††^	3.7 ± 0.5	5.3 ± 0.9^‡^	7.8 ± 1.4^‡^^,^^‡‡^
TAP-S (mm)	8.2 ± 0.9	9.0 ± 1.0*	9.8 ± 1.1*^,^**	7.6 ± 0.6	9.1 ± 0.7^†^	10.8 ± 0.9^†^^,^^††^	7.4 ± 0.4	9.1 ± 0.6^‡^	11.0 ± 0.7^‡^^,^^‡‡^
TAD-D (mm)	2.1 ± 0.3	2.3 ± 0.2*	2.7 ± 0.3*^,^**	2.1 ± 0.2	2.4 ± 0.2^†^	2.7 ± 0.4^†^^,^^††^	2.1 ± 0.3	2.4 ± 0.3^‡^	2.6 ± 0.4^‡^^,^^‡‡^
TAA-D (mm)	5.7 ± 1.1	7.1 ± 1.3*	9.0 ± 1.6*^,^**	5.5 ± 1.0	7.3 ± 1.2^†^	9.7 ± 1.4^†^^,^^††^	5.7 ± 1.1	7.3 ± 1.2^‡^	9.6 ± 1.4^‡^^,^^‡‡^
TAP-D (mm)	9.4 ± 0.9	10.4 ± 1.1*	11.3 ± 1.0*^,^**	9.1 ± 0.8	10.5 ± 0.9^†^	12.0 ± 0.8^†^^,^^††^	9.2 ± 0.9	10.5 ± 0.9^‡^	12.0 ± 0.7^‡^^,^^‡‡^
TAFS (%)	31.3 ± 10.5	21.1 ± 8.2*	17.2 ± 5.1*^,^**	23.3 ± 9.4	21.6 ± 8.7	17.8 ± 7.8	21.8 ± 8.8	21.9 ± 8.9	18.3 ± 3.5
TAFAC (%)	30.2 ± 12.3	27.1 ± 12.6	24.4 ± 10.4	34.4 ± 10.3	26.1 ± 9.9^†^	17.1 ± 6.3^†^^,^^††^	33.4 ± 10.4	26.1 ± 10.3^‡^	18.8 ± 5.6^‡^^,^^‡‡^

V_max_, maximum end-systolic right atrial volume; V_preA_, early diastolic pre-atrial contraction right atrial volume; V_min_, minimum end-diastolic right atrial volume; TASV, total atrial stroke volume; TAEF, total (right) atrial emptying fraction; PASV, passive (right) atrial stroke volume; PAEF, passive (right) atrial emptying fraction; AASV, active (right) atrial stroke volume; AAEF, active (right) atrial emptying fraction; TAD, tricuspid annular diameter; TAA, tricuspid annular area; TAP, tricuspid annular perimeter; TAFS, tricuspid annular fractional shortening; TAFAC, tricuspid annular fractional area change; S, end-systolic; D, end-diastolic.

* *p* < 0.05 vs. TAD-S_ _≤ 1.5 cm.

** *p* < 0.05 vs. 1.5 cm < TAD-S < 2.1 cm.

^†^ *p* < 0.05 vs. TAA-S_ _≤ 4.0 cm^2.^

^††^ *p* < 0.05 vs. 4.0 cm < TAA-S < 6.8 cm^2.^

^‡^ *p* < 0.05 vs. TAP-S_ _≤ 7.9 cm.

^‡‡^ *p* < 0.05 vs. 7.9 cm < TAP-S < 10.1 cm.

**Table 5:** Right atrial volumes and tricuspid annular parameters in different end-diastolic tricuspid annular groups.

	TAD-D_ _≤ 2.1 cm (*n* = 41)	2.1 cm < TAD-D < 2.7 cm (*n* = 80)	2.7 cm ≤ TAD-D (*n* = 23)	TAA-D_ _≤ 5.7 cm^2^ (*n* = 22)	5.7 cm < TAA-D < 8.9 cm^2^ (*n* = 97)	8.9 cm^2^ ≤ TAA-D (*n* = 25)	TAP-D_ _≤ 9.3 cm (*n* = 25)	9.3 cm < TAP-D < 11.7 cm (*n* = 95)	11.7 cm ≤ TAP-D (*n* = 24)
V_max_ (ml)	43.4 ± 12.6	49.7 ± 15.6*	55.8 ± 14.7*	36.4 ± 9.5	48.8 ± 13.7^†^	59.8 ± 16.5^†^^,^^††^	36.3 ± 9.2	49.9 ± 13.6^‡^	57.7 ± 17.5^‡^^,^^‡‡^
V_preA_ (ml)	31.9 ± 9.2	36.4 ± 10.6*	40.6 ± 13.1*	27.2 ± 7.8	35.9 ± 9.5^†^	42.9 ± 13.5^†^^,^^††^	27.8 ± 7.0	36.5 ± 10.0^‡^	41.5 ± 13.4^‡^^,^^‡‡^
V_min_ (ml)	24.4 ± 9.0	28.3 ± 9.4*	31.9 ± 10.5*	20.5 ± 6.7	27.9 ± 9.0^†^	33.7 ± 10.6^†^^,^^††^	21.5 ± 6.8	28.1 ± 8.9^‡^	32.8 ± 11.7^‡^^,^^‡‡^
TASV (ml)	19.0 ± 9.8	21.4 ± 10.0	23.8 ± 9.9	15.9 ± 8.2	21.0 ± 9.7^†^	26.2 ± 10.2^†^^,^^††^	14.8 ± 7.8	21.8 ± 9.4^‡^	24.9 ± 11.2^‡^
TAEF (%)	43.1 ± 15.1	42.2 ± 11.0	42.3 ± 13.4	42.6 ± 14.2	42.3 ± 12.7	42.2 ± 11.0	40.0 ± 13.3	43.1 ± 12.4	42.6 ± 12.5
PASV (ml)	11.5 ± 7.2	13.3 ± 8.6	15.2 ± 8.9	9.2 ± 7.2	13.0 ± 8.0^†^	16.9 ± 9.0^†^^,^^††^	8.6 ± 6.4	13.4 ± 7.8^‡^	16.2 ± 10.1^‡^
PAEF (%)	25.6 ± 12.3	25.5 ± 11.2	27.1 ± 13.0	24.2 ± 14.8	25.5 ± 11.2	28.1 ± 11.0	22.6 ± 12.5	26.2 ± 11.2	27.2 ± 12.6
AASV (ml)	7.5 ± 4.9	8.0 ± 4.5	8.6 ± 5.6	6.7 ± 4.2	8.0 ± 4.5	9.2 ± 5.9	6.2 ± 4.8	8.3 ± 4.6^‡^	8.7 ± 5.0
AAEF (%)	24.0 ± 13.9	22.2 ± 10.6	21.2 ± 10.4	24.2 ± 11.5	22.6 ± 11.9	21.0 ± 10.6	22.1 ± 13.7	23.1 ± 11.3	21.3 ± 10.3
TAD-S (mm)	1.6 ± 0.1	1.8 ± 0.2*	2.2 ± 0.3*^,^**	1.6 ± 0.1	1.8 ± 0.2^†^	2.2 ± 0.3^†^^,^^††^	1.6 ± 0.2	1.8 ± 0.3^‡^	2.0 ± 0.3^‡^^,^^‡‡^
TAA-S (mm)	4.4 ± 1.0	5.4 ± 1.1*	6.9 ± 1.7*^,^**	3.8 ± 0.5	5.3 ± 0.9^†^	7.3 ± 1.6^†^^,^^††^	3.9 ± 0.6	5.4 ± 1.1^‡^	6.8 ± 1.7^‡^^,^^‡‡^
TAP-S (mm)	8.2 ± 0.8	9.2 ± 1.0*	10.0 ± 1.1*^,^**	7.7 ± 0.7	9.0 ± 0.8^†^	10.3 ± 1.1^†^^,^^††^	7.9 ± 0.8	9.1 ± 0.9^‡^	10.1 ± 1.1^‡^^,^^‡‡^
TAD-D (mm)	2.0 ± 0.1	2.4 ± 0.1*	2.8 ± 0.2*^,^**	2.0 ± 0.2	2.3 ± 0.2^†^	2.7 ± 0.3^†^^,^^††^	2.1 ± 0.2	2.4 ± 0.3^‡^	2.5 ± 0.4^‡^^,^^‡‡^
TAA-D (mm)	6.0 ± 1.1	7.4 ± 1.2*	9.2 ± 1.4*^,^**	5.0 ± 0.5	7.2 ± 0.8^†^	9.8 ± 1.1^†^^,^^††^	5.3 ± 0.7	7.3 ± 1.0^‡^	9.5 ± 1.4^‡^^,^^‡‡^
TAP-D (mm)	9.8 ± 1.1	10.5 ± 1.0*	11.5 ± 1.0*^,^**	8.8 ± 0.6	10.4 ± 0.8^†^	12.0 ± 0.6^†^^,^^††^	8.8 ± 0.5	10.4 ± 0.7^‡^	12.2 ± 0.4^‡^^,^^‡‡^
TAFS (%)	18.0 ± 7.3	22.9 ± 9.4*	22.5 ± 7.6*	21.6 ± 7.1	21.8 ± 9.5	19.8 ± 7.1	22.8 ± 7.3	21.1 ± 9.4	21.3 ± 7.7
TAFAC (%)	26.9 ± 10.6	26.5 ± 10.7	25.5 ± 10.4	24.8 ± 9.8	26.9 ± 10.8	26.3 ± 10.5	26.5 ± 9.9	25.6 ± 10.8	29.5 ± 10.4

V_max_, maximum end-systolic right atrial volume; V_preA_, early diastolic pre-atrial contraction right atrial volume; V_min_, minimum end-diastolic right atrial volume; TASV, total atrial stroke volume; TAEF, total (right) atrial emptying fraction; PASV, passive (right) atrial stroke volume; PAEF, passive (right) atrial emptying fraction; AASV, active (right) atrial stroke volume; AAEF, active (right) atrial emptying fraction; TAD, tricuspid annular diameter; TAA, tricuspid annular area; TAP, tricuspid annular perimeter; TAFS, tricuspid annular fractional shortening; TAFAC, tricuspid annular fractional area change; S, end-systolic; D,  end-diastolic.

* *p* < 0.05 vs. TAD-D_ _≤ 2.1 cm.

** *p* < 0.05 vs. 2.1 cm < TAD-D < 2.7 cm.

^†^ *p* < 0.05 vs. TAA-D_ _≤ 5.7 cm^2.^

^††^ *p* < 0.05 vs. 5.7 cm < TAA-D < 8.9 cm^2.^

^‡^ *p* < 0.05 vs. TAP-D_ _≤ 9.3 cm.

^‡‡^ *p* < 0.05 vs. 9.3 cm < TAP-D < 11.7 cm.

### Dilation of TA and TA functional properties

With increasing end-systolic TA dimensions, TA functional properties showed simultaneous decrease, but in different degrees. With increasing end-diastolic TAD-D, TAFS increased, TAFAC remained unchanged. With increasing end-diastolic TAA-D and TAP-D, TA functional properties did not show significant changes (Tables 4, 5).

### Correlations and regression analysis

V_max_ correlated with TAD-D (r = 0.30, *p* < 0.01), TAA-D (r = 0.54, *p* < 0.01), TAA-P (r = 0.50, *p* < 0.01), TAD-S (r = 0.39, *p* < 0.01), TAA-S (r = 0.55, *p* < 0.01) and TAA-P (r = 0.49, *p* < 0.01). Similarly, V_preA_ showed correlations with TAD-D (r = 0.25, *p* < 0.01), TAA-D (r = 0.49, *p* < 0.01), TAA-P (r = 0.45, *p* < 0.01), TAD-S (r = 0.40, *p* < 0.01), TAA-S (r = 0.58, *p* < 0.01) and TAA-P (r = 0.49, *p* < 0.01). V_min_ correlated with TAD-D (r = 0.54, *p* < 0.01), TAA-D (r = 0.48, *p* < 0.01), TAA-P (r = 0.41, *p* < 0.01), TAD-S (r = 0.37, *p* < 0.01), TAA-S (r = 0.54, *p* < 0.01) and TAA-P (r = 0.46, *p* < 0.01), as well.

The logistic regression analysis identified presence of increased V_max_ as an independent predictor of TAA-D [hazard ratio (HR) 1.75, 95% CI of HR: 1.18 to 3.33, *p* < 0.05]. Similarly, dilated TAA-D had an independent predictive value for V_max_ [hazard ratio (HR) 1.80, 95% CI of HR: 1.15 to 3.27, *p* < 0.05].

### Feasibility of 3DSTE-derived RA and TA measurements

During evaluations, 94 subjects were excluded due to inferior image quality from the total of 238 subjects, therefore the overall feasibility of simultaneous 3DSTE-derived RA and TA quantifcations was 144 out of 238 (61% overall feasibility).

### Reproducibility of 3DSTE-derived RA and TA assessments

3DSTE-derived end-diastolic and end-systolic TA dimensions and RA volumes respecting cardiac cycle were measured twice by the same observer (intraobserver agreement) and by two independent observers (interobserver agreement). The values were expressed as mean ± SD together with corresponding ICCs, the results are presented in Table 6.

**Table 6:** Intra- and interobserver variability for three-dimensional speckle-tracking echocardiography-derived tricuspid annular dimensions and right atrial volumes.

	Intraobserver agreement		Interobserver agreement	
	Mean ± 2SD difference in values obtained by 2 measurements of the same observer	ICC between measurements of the same observer	Mean ± 2SD difference in values obtained by 2 observers	ICC between independent measurements of 2 observers
End-diastolic TAD	0.02 ± 0.21 cm	0.96 (*p* < 0.0001)	0.03 ± 0.15 cm	0.96 (*p* < 0.0001)
End-diastolic TAA	−0.04 ± 1.14 cm^2^	0.95 (*p* < 0.0001)	0.02 ± 0.56 cm^2^	0.96 (*p* < 0.0001)
End-diastolic TAP	−0.03 ± 0.71 cm	0.95 (*p* < 0.0001)	−0.11 ± 0.58 cm	0.96 (*p* < 0.0001)
End-systolic TAD	−0.03 ± 0.32 cm	0.96 (*p* < 0.0001)	0.02 ± 0.44 cm	0.96 (*p* < 0.0001)
End-systolic TAA	−0.04 ± 0.31 cm^2^	0.95 (*p* < 0.0001)	−0.05 ± 0.65 cm^2^	0.97 (*p* < 0.0001)
End-systolic TAP	0.07 ± 0.55 cm	0.96 (*p* < 0.0001)	0.04 ± 0.59 cm	0.97 (*p* < 0.0001)
Vmax	1.2 ± 6.3 ml	0.96 (*p* < 0.0001)	1.0 ± 5.2 ml	0.95 (*p* < 0.0001)
VpreA	−1.5 ± 8.6 ml	0.87 (*p* < 0.0001)	−1.5 ± 8.3 ml	0.90 (*p* < 0.0001)
Vmin	0.8 ± 5.1 ml	0.94 (*p* < 0.0001)	0.9 ± 4.6 ml	0.94 (*p* < 0.0001)

ICC, interclass correlation coefficient; TAD, tricuspid annular diameter; TAA, tricuspid annular area; TAP, tricuspid annular perimeter; SD, standard deviation; V_max_, maximum end-systolic right atrial volume; V_preA_, early diastolic pre-atrial contraction right atrial volume; V_min_, minimum end-diastolic right atrial volume.

## Discussion

Components of the TV or right atrioventricular valve include fibrous TA, anterior, posterior and septal leaflets, papillary muscles and tendinous cords, which interact with RA and RV during the cardiac cycle. When TV opens in diastole, it helps correct one-way blood flow from the RA to the RV, while in systole, it closes to prevent backflow or regurgitation from the RV into the RA. The normal TA is a dynamic structure with a saddle-shape like MA, its dilation is accompanied with a more circular and planar shape (16, 17). Tricuspid regurgitation is organic only in 10–15% of cases (18), most of the cases show functional regurgitation (FTR) due to distorted RV, subvalvular apparatus, or TA, with structurally normal tricuspid leaflets (19, 20). FTR is mostly due to LV dysfunction, aortic or mitral valve disease or pulmonary vascular or interstitial disorders and is accompanied with consequent pulmonary hypertension (PH). If FTR is secondary to TA dilation and leaflet tethering and associated to RV dilation and/or dysfunction, it has been called as “classical” or ventricular form of FTR for a long time. In case of absent PH or left heart disorders, FTR was called as idiopathic tricuspid regurgitation previously, which is related to age and atrial fibrillation (AF) (19, 20). Recently, a new distinct entity has been created called as atrial FTR, which can be found in AF patients, in which RA enlargement and dysfunction result in TA dilation, leaflet malcoaptation and loss of TA spinchter-like function (21). Therefore, RA rather than RV dilation is more important to determine TA dilation and development of FTR in such cases (5). Similarly, following heart transplantation, overall RA diameter and native recipient RA diameter were found to be a risk factor for TA distension, which is reported to be a causative factor for the most common type of TV dysfunction (22).

Routine non-invasive TV assessment is based on 2D Doppler echocardiography, but results with 3D echocardiography are also available (23). 3DSTE has been demonstrated to be capable not only of performing RA chamber quantifications (14), but for determination of atrioventricular annular dimensions and its sphincter-like functional properties at the same time using the same 3D acquired datasets from the transthoracic window (15). Age- and gender-dependency and normal reference values for 3DSTE-derived RA and TA assessments were also determined with considerable intra- and interobserver agreements (14, 15). Therefore, 3DSTE seems to be an optimal option for simultaneous assessment of TA and RA for (patho)physiologic studies. According to present findings, although feasibility of simultaneous assessment of 3DSTE-derived RA volumes and TA dimensions proved to be limited, however, reproducibility was found to be acceptable in those cases where the measurements were feasible.

There can be a question whether associations between RA size and TA dimensions exist before FTR develops in healthy adults in sinus rhythm. It is known that with increasing left atrial volumes, mitral annulus dilated and became functionally impaired under healthy circumstances (24). The present study serves as an analogy for this by examining what happens in the right side of the heart under similar conditions. Results show similar findings demonstrating strong associations in case of dilation of TA in the presence of higher RA volumes. Moreover, dilation of TA and enlargement of RA predicted each other, as well. It is more important in the context of recent findings from the MAGYAR-Healthy Study, where strong associations between RA radial strains and end-diastolic TA area could also be detected in healthy subjects without FTR (9). However, further clinical studies are warranted to confirm our findings in a larger population or in different pathologic states and to demonstrate their possible clinical role in patient management. Moreover, long-term follow-up could confirm predictive role of TA/RA dilation on the development of AF and/or FTR.

### Limitations

Several important limitations have arisen during assessments:

– 2D echocardiography still allows TA assessment with a better image quality as compared to 3DSTE.
– The results would have been much more convincing if the measurement results had been validated against those measured with the Tomtect software. This topic could even be the subject of a subsequent study.
– Although TA has a special 3D saddle-shape, only its 2D-projected image was determined.
– The study did not aim to compare 2D echocardiography vs. 3DSTE in the assessment of TA.
– Speckle-tracking analysis of TA functionality was not purposed, as well.
– FTR was excluded by visual assessment, more advanced quantification method was not applied during evaluations.

## Conclusions

3DSTE is suitable for non-invasive assessment of TA dimensions and RA volumes at the same time using the same 3D echocardiographic dataset. Significant associations between TA size and RA volumes exist in healthy circumstances. Strong associations in case of dilation of TA in the presence of higher RA volumes could partly explain FTR later developing in subjects in sinus rhythm.

## Data Availability

The datasets presented in this article are not readily available due to local restrictions. Requests to access the datasets should be directed to nemes.attila@med.u-szeged.hu.

## References

[1] Lang RM Badano LP Mor-Avi V Afilalo J Armstrong A Ernande L Recommendations for cardiac chamber quantification by echocardiography in adults: an update from the American society of echocardiography and the European association of cardiovascular imaging. Eur Heart J Cardiovasc Imaging. (2015) 16:233–70. 10.1093/ehjci/jev014 25712077

[2] Rudski LG Lai WW Afilalo J Hua L Handschumacher MD Chandresakaran K Guidelines for the echocardiographic assessment of the right heart in adults: a report from the American Society of Echocardiography endorsed by the European Association of Echocardiography, a registered branch of the European Society of Cardiology, and the Canadian Society of Echocardiography. J Am Soc Echocardiogr. (2010) 23:685–713. 10.1016/j.echo.2010.05.010 20620859

[3] Muraru D Hahn RT Soliman OI Faletra FF Basso C Badano LP. 3-Dimensional echocardiography in imaging the tricuspid valve. JACC Cardiovasc Imaging. (2019) 12:500–15. 10.1016/j.jcmg.2018.10.035 30846124

[4] Tadic M. The right atrium, a forgotten cardiac chamber: an updated review of multimodality imaging. J Clin Ultrasound. (2015) 43:335–45. 10.1002/jcu.22261 25732678

[5] Guta AC Badano LP Tomaselli M Mihalcea D Bartos D Parati G The pathophysiological link between right atrial remodeling and functional tricuspid regurgitation in patients with atrial fibrillation: a three-dimensional echocardiography study. J Am Soc Echocardiogr. (2021) 34:585–594.e1. 10.1016/j.echo.2021.01.004 33440232

[6] Muraru D Addetia K Guta AC Ochoa-Jimenez R Genovese D Veronesi F Right atrial volume is a major determinant of tricuspid annulus area in functional tricuspid regurgitation: a three-dimensional echocardiography study. Eur Heart J Cardiovasc Imaging. (2021) 22:660–9. 10.1093/ehjci/jeaa286 33387441

[7] Florescu DR Muraru D Florescu C Volpato V Caravita S Perger E Right heart chambers geometry and function in patients with the atrial and the ventricular phenotypes of functional tricuspid regurgitation. Eur Heart J Cardiovasc Imaging. (2022) 23:930–40. 10.1093/ehjci/jeab211 34747460

[8] Muraru D Gavazzoni M Heilbron F Mihalcea DJ Guta AC Radu N Reference ranges of tricuspid annulus geometry in healthy adults using a dedicated three-dimensional echocardiography software package. Front Cardiovasc Med. (2022) 9:1011931. 10.3389/fcvm.2022.1011931 36176994 PMC9513148

[9] Nemes A Kormányos Á Ruzsa Z Achim A Ambrus N Lengyel C. Three-dimensional speckle-tracking echocardiography-derived tricuspid annular dimensions and right atrial strains in healthy adults-is there a relationship? (insights from the MAGYAR-Healthy Study). J Clin Med. (2023) 12:4240. 10.3390/jcm12134240 37445275 PMC10342321

[10] Urbano-Moral JA Patel AR Maron MS Arias-Godinez JA Pandian NG. Three-dimensional speckle-tracking echocardiography: methodological aspects and clinical potential. Echocardiography. (2012) 29:997–1010. 10.1111/j.1540-8175.2012.01773.x 22783969

[11] Ammar KA Paterick TE Khandheria BK Jan MF Kramer C Umland MM Myocardial mechanics: understanding and applying three-dimensional speckle tracking echocardiography in clinical practice. Echocardiography. (2012) 29:861–72. 10.1111/j.1540-8175.2012.01712.x 22591237

[12] Muraru D Niero A Rodriguez-Zanella H Cherata D Badano L. Three-dimensional speckle-tracking echocardiography: benefits and limitations of integrating myocardial mechanics with three-dimensional imaging. Cardiovasc Diagn Ther. (2018) 8:101–17. 10.21037/cdt.2017.06.01 29541615 PMC5835646

[13] Lancellotti P Tribouilloy C Hagendorff A Popescu BA Edvardsen T Pierard LA Recommendations for the echocardiographic assessment of native valvular regurgitation: an executive summary from the European Association of Cardiovascular Imaging. Eur Heart J Cardiovasc Imaging. (2013) 14:611–44. 10.1093/ehjci/jet105 23733442

[14] Nemes A Kormányos Á Domsik P Kalapos A Ambrus N Lengyel C. Normal reference values of three-dimensional speckle-tracking echocardiography-derived right atrial volumes and volume-based functional properties in healthy adults (Insights from the MAGYAR-Healthy Study). J Clin Ultrasound. (2020) 48:263–8. 10.1002/jcu.22795 31737908

[15] Nemes A Kormányos Á Rácz G Ruzsa Z Ambrus N Lengyel C. Normal reference values of tricuspid annular dimensions and functional properties in healthy adults using three-dimensional speckle-tracking echocardiography (insights from the MAGYAR-Healthy Study). Quant Imaging Med Surg. (2023) 13:121–32. 10.21037/qims-22-88 36620137 PMC9816735

[16] Ton-Nu TT Levine RA Handschumacher MD Dorer DJ Yosefy C Fan D Geometric determinants of functional tricuspid regurgitation: insights from 3-dimensional echocardiography. Circulation. (2006) 114:143–9. 10.1161/CIRCULATIONAHA.106.611889 16818811

[17] Addetia K Muraru D Veronesi F Jenei C Cavalli G Besser SA 3-dimensional echocardiographic analysis of the tricuspid annulus provides new insights into tricuspid valve geometry and dynamics. JACC Cardiovasc Imaging. (2019) 12:401–12. 10.1016/j.jcmg.2017.08.022 29153573

[18] Muraru D Addetia K Jarjour F Lang RM Badano LP. Organic tricuspid regurgitation. In: Badano LP Lang RM Muraru D, editors. Textbook of three-dimensional echocardiography. Cham, Switzerland: Springer (2019). p. 271–83

[19] Gual-Capllonch F Cediel G Ferrer E Teis A Juncà G Vallejo N Sex-related differences in the mechanism of functional tricuspid regurgitation. Heart Lung Circ. (2021) 30:e16–22. 10.1016/j.hlc.2020.06.018 32771383

[20] Badano LP Muraru D Enriquez-Sarano M. Assessment of functional tricuspid regurgitation. Eur Heart J. (2013) 34:1875–85. 10.1093/eurheartj/ehs474 23303656

[21] Florescu DR Muraru D Volpato V Gavazzoni M Caravita S Tomaselli M Atrial functional tricuspid regurgitation as a distinct pathophysiological and clinical entity: no idiopathic tricuspid regurgitation anymore. J Clin Med. (2022) 11:382. 10.3390/jcm11020382 35054074 PMC8781398

[22] Urbanowicz T Michalak M Kociemba A Straburzyńska-Migaj E Katarzyński S Grajek S Predictors of tricuspid valve anulus dilation in a heart recipient population. Transplant Proc. (2016) 48:1742–5. 10.1016/j.transproceed.2016.01.093 27496483

[23] Anwar AM Geleijnse ML Soliman OII McGhie JS Frowijn R Nemes A Assessment of normal tricuspid valve anatomy in adults by real-time three-dimensional echocardiography. Int J Cardiovasc Imaging. (2007) 23:717–24. 10.1007/s10554-007-9210-3 17318363 PMC2048827

[24] Nemes A Kormányos Á Ambrus N Lengyel C. Associations between mitral annular and left atrial volume changes in healthy adults—Detailed analysis from the three-dimensional speckle-tracking echocardiographic MAGYAR-Healthy Study. Rev Cardiovasc Med. (2022) 23:194. 10.31083/j.rcm2306194 PMC11273884 39077185

